# Distribution of IL-28B genotypes in patients with hepatitis C and healthy individuals in Jahrom city 

**Published:** 2015

**Authors:** Seyed Dawood Mousavi Nasab, Rasoul Baharlou, Ahmad Piroozmand, Hadi Toghyani, Enayatollah Shadmand, Hadi Fazel, Kaveh Sadeghi, Seyed Mohammad Ali Hashemi, Mohammad Reza Shokouh, Abulfazl Gheshlaghi, Nayeb Ali Ahmadi, Abbas Ahmadi Vasmehjani

**Affiliations:** 1 *Department of Microbiology, Faculty of Medicine, Kashan University of Medical Sciences, Kashan, Iran*; 2 *Autoimmune Diseases Research Center, School of Medicine, Kashan University of Medical Sciences, Kashan, Iran*; 3 *Department of Microbiology, Jahrom University of Medical Sciences, Jahrom, Iran*; 4 *Department of Virology, School of Public Health, Tehran University of Medical Sciences, Tehran, Iran*; 5 *Proteomics Research Center, and Department of Medical Lab Technology, Faculty of Paramedical Sciences, Shahid Beheshti University of Medical Sciences, Tehran, Iran*

**Keywords:** Polymorphism, Hepatitis C; IL-28B Genotype, Jahrom

## Abstract

**Aim:**

The purpose of this study was to compare the distribution of interleukin (IL)-28B genotypes between Iranian healthy individuals and patients with chronic hepatitis C based on the genotype.

**Background:**

Polymorphisms in the region of IL-28B gene have been identified as the strongest genetic pretreatment predictor of sustained virological response (SVR) in hepatitis C infection.

**Patients and methods:**

In this study, 147 patients with chronic hepatitis C and 80 healthy individuals were included. The IL-28B rs12979860 and rs8099917 polymorphisms were genotyped by PCR-RFLP method and the frequency of IL-28B polymorphisms with respect to HCV genotypes was also determined.

**Results:**

The frequencies of rs12979860 TT, CC and CT genotypes in the chronic hepatitis C patients and healthy individuals were as follows: 10.8% vs. 11.3%, 38.7% vs. 46.2% and 50.3% vs. 42.5%. Also, the frequencies of rs8099917 TT, GG and GT genotypes in the chronic hepatitis C patients was 61.9%, 6.1% and 32% and in controls was 47.5%, 11.2% and 41.3%. The differences in the distribution of rs12979860 genotypes and alleles between HCV genotype 1 and HCV genotype 3a infected patients were statistically significant.

**Conclusion:**

The rs12979860 C allele is the favorable allele for the spontaneous clearance of HCV. It seems that the impact of IL-28B polymorphism on the spontaneous clearance of HCV genotype 3 is more prominent than HCV genotype 1, which results in the observation of higher rs12979860 C allele frequency in chronic hepatitis C patients with HCV genotype 3 than HCV genotype 1.

## Introduction

 Based on the World health organization (WHO) reports, about 3% of the world population is infected with hepatitis C virus (HCV) that is a major cause of chronic viral hepatitis along with the hepatitis B virus (1). A large proportion (50-90%) of the patients infected with HCV is destined to suffer from chronic infection, which is associated with variable degrees of hepatic inflammation and fibrosis progression. Spontaneous clearance occurs in 20%-25% of patients with acute hepatitis C infection (2). Approximately 10%-40% of patients with chronic HCV infection will develop into cirrhosis. Hepatocellular carcinoma occurs in this population at an estimated incidence of 1%-5%. 

Up till now the current standard care for the treatment of patients with hepatitis C is Pegylated interferon alpha (PEG-IFN) in combination with Ribavirin (RBV). However, this strategy does not eliminate HCV in approximately 40%-50% of infected patients with HCV genotype 1 or 4 (3-5) and 20%-30% in HCV genotype 3 infected patients (4). Recently, two new drugs for hepatitis C, including boceprevir and telaprevir have been approved by FDA. Boceprevir and telaprevir promise a better chance of a cure and administrate together with current standard therapy, however they are very expensive and have potentially serious side effects (6). 

In addition to viral genotype and host immunity (7, 8), host genetic factors recently have been identified to influence treatment responsiveness in patients with chronic hepatitis C. Among host factors, many single nucleotide polymorphisms (SNPs) that associated with outcome of therapy have been identified. Different studies have shown SNPs near the IL28B gene on chromosomes 19 that encodes interferon lambda. These SNPs have been strongly associated with response to Peg-IFN and RBV combination therapy in HCV genotype 3a and 1–infected patients (9-11). Studies have revealed that rs12979860 and rs8099917 SNPs are associated with a SVR to HCV treatment (2, 12, 13). 

The IL-28B rs12979860 CC and rs8099917 TT genotypes are associated with a higher probability of treatment-induced viral clearance than IL-28B rs12979860 T and rs8099917 G alleles. Distribution of IL-28B genotypes is varied in various races. Also, the association of IL-28B SNPs and treatment-related response was virtually independent of the ethnicity of the study population (14, 15). In Asia, interferon-based therapy such as rs12979860 and rs8099917 SNPs seem appropriate for most patients with genotypes 1 and 2. Interferon-based therapy may limit the usefulness of IL-28 SNP in therapy response prediction; therefore the determination of frequency of IL-28B SNPs in HCV patients and healthy individuals with similar races is essential. (16, 17). Another finding in recent studies was the association between HCV genotype and IL-28B polymorphisms in which the frequency of IL-28B favorable alleles was different in the various HCV genotypes in HCV infected patients (18-20). In the southern part of Iran few data are available on distribution of IL-28B genotypes in chronic hepatitis C. Therefore, in the current study, we aimed to determine the distribution of IL-28B rs12979860 and rs8099917 genotypes in HCV infected and healthy individuals as well as its association with HCV genotype. 

## Patients and Methods

***Study Population***

Present cross-sectional study was designed, using 147 positive HCV patients who were registered at the Honary Medical Clinic Centre in Jahrom, a town in the southern part of Iran, between September 2012 and February 2013. Patients provided signed informed consent prior to participation. The patients with chronic infection were anti-HCV antibody positive and had positive plasma HCV RNA for more than 6 months. However, the patients with acute HCV infection were diagnosed based on the first detection of HCV RNA and elevated ALT. These patients have not HCV antibody and HCV RNA in sampling time. Patients who spontaneously clear their infection and patients with human immunodeficiency virus (HIV) infection were excluded from the study. A random sample of healthy volunteers (n=80), anti-HCV antibody, HIV and HBsAg negative, were enrolled for IL-28B genotyping, as controls. The patients co-infected with hepatitis B virus (HBV) or human immunodeficiency virus (HIV) (tested by standard serological tests (HBs antigen, HIV-1/2 antibodies) were excluded from this study. 

***cDNA Synthesis and HCV Genotyping***

**Figure 1:**
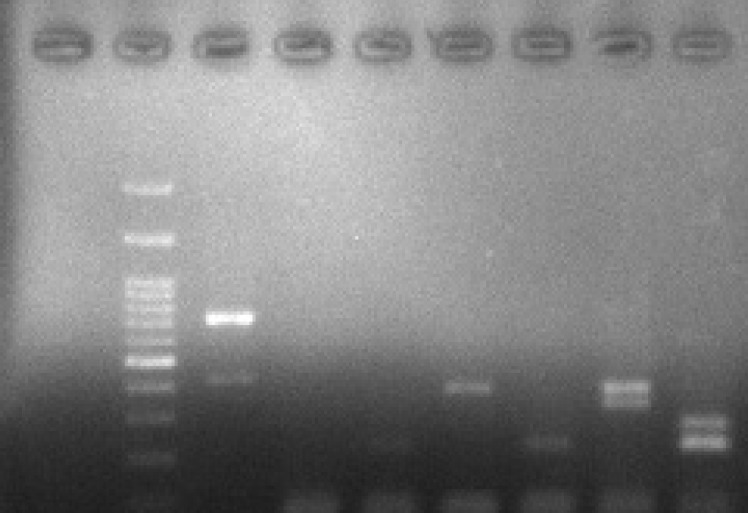
Typical electrophoresis patterns of PCR products from different HCV genotypes. Patterns of four serum samples left to right are shown for containing HCV genotypes 1a (lane 5), 1b (lane 2), 3a (lanes 4 and 6), Mix genotype 2/3a (lane 8) and 1a/1b (line 7). The patterns of molecular size markers (GeneRuler™ 100 bp Plus DNA Ladder, ready-to-use; Fermentas UAB, Vilnius, Lithuania) are indicated on lane 1. The lengths of PCR products were 338 bp for genotype 1a, 398 bp for 1b, 286 bp for 2 and 227 bp for 3a

According to the manufacturer's instructions, RNA was extracted from 100 µl of plasma, using the AccuPrep Viral RNA Extraction Kit (Bioneer, South Korea). The final volume, in the reaction for synthesis of cDNA, was 20 µl, including 5 µl RNA, 1 µl random hexamer (Fermentas GmbH, Germany), 6.5 µl of diethylpyrocarbonate (DEPC) treated water, 4 µl reverse transcriptase reaction buffer 5x, 2 µl dNTP, 10 mM stock, (Fermentase GmbH, Germany), 0.5 µl RNase Inhibitor (Fermentas GmbH, Germany) and 200 IU of Moloneymurine leukemia virus reverse transcriptase (Fermentas GmbH, Germany), which was then incubated at 65°C for 5 minutes, 25°C for 10 minutes, 42°C for 60 minutes and 70°C for 10 minutes. HCV genotype was determined using type-specific primers as described previously (21). Each 25 µl reaction mixture contained 5 µl of template, 2.5 µl of 10X reaction buffer (Applied Biosystems, Foster City, CA), 1.5 mM MgCl2 (AB), 0.5 mM of each dNTPs (AB), HCV-specific primers, and AmpliTaq DNA polymerase (AB). The amount of specific-HCV primers in each reaction based on the type of primer was different. Then, 2 µL of this cDNA was amplified for 40 cycles with the following parameters: a preliminary 20 cycles of amplification at 94°C for 1 min (denaturing), 45°C for 1 min (annealing), and 72°C for 1 min (extension), followed by 20 additional cycles of 94°C for 1 min, 60°C for 1 min, and 72°C for 1 min. For the second-round of PCR, 0.5 ml of first-round PCR product was amplified for 30 cycles; each cycle consisted of 94°C for 1 min, 62°C for 45 s, and 72°C for 1 min. Finally, the analysis of results was performed on the basis of the presence or absence of specific bands of amplified DNA in the 2% agarose gel (Figure 1).

**Figure 2:**
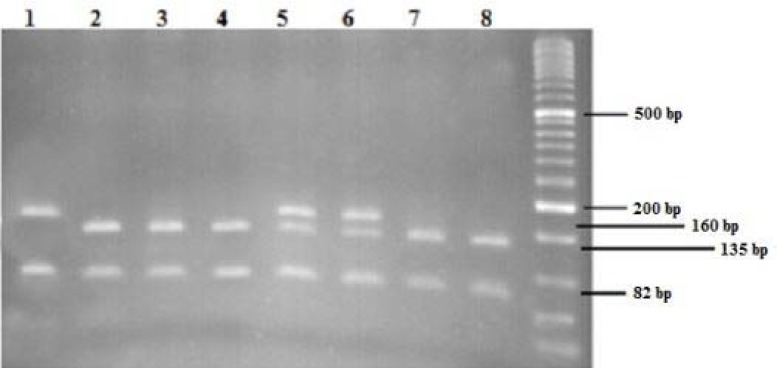
rs12979860 PCR-RFLP genotyping. Digestion of this product with Bstu І in CC individuals produced 3 fragments of 135bp, 82bp, 25bp; 4 fragments of 160bp, 135bp, 82bp , 25bp in CT individuals; and 2 fragment of 160bp , 82bp in TT individuals. Lanes no. 1 was genotyped TT, 2, 3, 4, 7 & 8 were genotyped CC, lane no. 5 & 6 were genotyped CT

***IL-28B genotyping***

Blood was collected into EDTA tubes. Genomic DNA was extracted using the QIAamp DNA Blood Mini Kit (Qiagen, Hilden, Germany) according to the manufacturer’s instructions. DNA was quantified using a NanoDrop spectrophotometer. Genotyping of the rs12979860 and rs8099917 SNPs was performed based on previous studies (22). The PCR was performed using Accupower® PCR PreMix (Bioneer, South Korea) with a 20 µl reaction tube type. The polymorphisms were genotyped using PCR with specific primers as follows: 10 ng of genomic DNA was amplified using 10 pmol of primers IL-28B rs8099917, 5΄-CCACTTCTGGAACAAATCGTC-3΄ and 5΄-GATACGCTATAATTAAAGATGTGGGA-3΄ was amplified in 32 cycles at 94°C for 30 s, 56°C for 45 s and 72°C for 1 min ; and in the case of IL-28B rs12979860, 10 ng of genomic DNA was amplified using 10 pmol of primers 5΄-GCTTATCGCATACGGCTAGG-3΄ and 5΄-AGGCTCAGGGTCAATCACAG-3΄ was amplified in 32 cycles at 94°C for 30 s, 56°C for 45 s and 72°C for 1 min. The amplified products were sequenced and analyzed using Sequence Detection System 2.3 Software. Genotyping was confirmed by PCR-RFLP method (Figure 2). 

***Statistical Analysis***

Statistical differences between groups were determined using the Chi-square test. Logistic regression analyses were performed to identify independent predictors of IL-28B polymorphism. A 2-sided P value of less than 0.05 was considered statistically significant. Statistical analysis was performed using SPSS version 19.0. 

## Results

***Groups Characteristics***

Demographic of both groups are shown in Table 1. Distribution of HCV genotypes among 147 patients with hepatitis C is demonstrated in Figure 1. Subtype 3 was the most common genotype in plasma (57.7%) (Figure 1). Subtype 3 was also the most frequent genotype in patients over the age of 40 than patients who were less than 40 years old (47.3% versus 46.5%). However, there was no difference between the mean age of the subgroup of patients with hepatitis C. Infection rates in males were higher than female but there was no significant difference for the rate of HCV infection among male and female groups.

***Distribution of IL-28B Genotypes and alleles in Patients with hepatitis C and healthy individuals***

The distribution of IL-28B rs12979860 and rs8099917 genotypes as well as alleles in patients with hepatitis C and healthy individuals are reported in Table 1. The distribution of CT and CC genotypes in IL-28B rs12979860 SNPs was higher than other type in patients with hepatitis C and healthy individuals respectively. The distribution of the TT genotype in IL-28B rs8099917 SNPs was higher than other types in patients with hepatitis C and healthy individuals respectively. The differences in the distribution of IL-28B genotypes between patients with hepatitis C and healthy individuals related to rs12979860 and rs8099917 were not statistically significant (P=0.36 and P=0.14 respectively). The distribution of the C allele in IL-28B rs12979860 SNPs was higher than the T allele in patients with hepatitis C and healthy individuals respectively. The distribution of T allele in IL-28B rs8099917 SNPs was higher than G allele in patients with hepatitis C and healthy individuals respectively. The differences in the distribution of IL-28B rs12979860 alleles between patients with hepatitis C and healthy individuals were statistically significant but this difference in rs8099917 alleles were not significant (Table 1).

**Table 1:** Characteristics of demographic and distribution of IL-28 genotypes and alleles in both groups

Variables	HCV (%)	Control (%)	*p* value
Age	41.8±11	43.2±1.6	0.94
Gender Female Male	60 (40.8%) 87 (59.1%)	34 (46.2%) 46 (53.8%)	0.18
HCV Genotype 3a 1a Mixed	79 (53.7%) 60 (40.8%) 8 (5.4%)		
rs8099917 genotypes GG GT TT	9 (6.1%) 47 (32%) 91 (61.9%)	9 (11.2%) 33 (41.3%) 38 (47.5%)	0.14
allele frequency G T	33 (22.1%) 114 (77.9%)	26(31.9%) 54 (68.1%)	0.41
rs12979860 genotypes CC CT TT	57 (38.7%) 74 (50.3%) 16 (10.8%)	37 (46.2%) 34 (42.5%) 9 (11.3%)	0.36
allele frequency C T	94 (63.9%) 53 (36.1%)	54 (67.5%) 26 (32.5%)	0.03

GT: Genotype; MGT: Mix genotypes

***Distribution of IL-28B genotypes and alleles in patients with hepatitis C based on each genotype and comparison with health individuals***

The differences in the distribution of IL-28B rs12979860 genotypes and alleles between patients with hepatitis C different genotypes were statistically significant (P=0.02, P=0.03) (Table 2). Frequency of IL-28B rs12979860 CT genotype in HCV genotype 3a infected patients was 23.7% and 37% higher than HCV genotype 1a infected patients and healthy individuals. Also, distribution of IL-28B rs12979860 TT genotypes in HCV genotype 1a infected patients was 11.6% higher than HCV genotype 3a infected patients and this rate was 8.3% lower than healthy individuals. We did not find a significant difference in the frequency of rs8099917 genotypes and alleles between HCV genotype 3a and HCV genotype 1 infected patients and healthy individuals (P=0.56 and P=0.35, respectively) (Table 2). Also frequencies of GT and TT in HCV genotype 3a infected patients were 0.4% higher than 1 infected patients and 1.4% lower than these patients. The differences in the distribution of rs12979860 genotypes and alleles between HCV genotype 1 and HCV genotype 3a infected patients were statistically significant. However, the difference in the distribution of rs8099917 genotypes based on HCV genotypes was not statistically significant (Table 2). There was no significant difference between the distribution of IL-28B rs12979860 and rs8099917 genotypes, and alleles in mixed genotypes (multiple genotypes) infected patients with healthy individuals (Data not shown).

**Table 2:** Comparison of distribution of IL-28B genotypes and alleles respected to HCV genotypes

Variables	GT3 (n=79)	GT1 (n=60)	MGT (n=8)	P	OR	95% CI
Gender Male Female	41 (48.1%) 38 (51.8%)	36 (60%) 24 (40%)	3 (37.5%) 5 (62.5%)	0.24		
Age (mean± SD)	43.1±11.3	41.5±1.8	40.1±1.7	0.32		
rs8099917 genotypes GG GT TT	6 (7.6%) 24 (30.4%) 49 (62%)	4 (6.6%) 18 (30%) 38 (63.4%)	1 (12.5%) 2 (25%) 5 (62.5%)	0.56	0.5	(0.4-1.4)
allele frequency G T	18 (22.8%) 61 (77.2%)	13 (21.6%) 47 (88.4%)	2 (25%) 6 (75%)	0.35	0.8	(0.4-2.05)
rs12979860 genotypes CC CT TT	26 (32.9%) 49 (62%) 4 (5.1%)	27 (45%) 23 (38.3%) 10 (16.7%)	4 (50%) 2 (25%) 2 (25%)	0.02	1.4	(0.5-1.5)
allele frequency C T	51 (64.5%) 28 (35.5%)	39 (65%) 21 (35%)	5 (62.5%) 3 (37.5%)	0.03	2.95	(0.95-1.16)

GT: Genotype; MGT: Mix genotypes; CI: confidence interval; OR, odds ratio.

***Distribution of IL-28B Genotypes in Patients with hepatitis C based on liver disease status***

Among patients with hepatitis C, 91 (61.9%) had chronic hepatitis C and 56 (38.1%) had acute infection. Distribution of IL-28B genotypes based on liver disease status, acute and chronic HCV-infected patients, was determined. The distribution of IL-28B rs12979860 genotypes among the patients with acute hepatitis C was as the following: 36 (64.3%) were CC, 20 (35.7%) were CT. Among chronic hepatitis C patients, 22 (24.2%) were CC, 57 (62.6%) were CT, and 12 (13.2%) were TT. The distribution of IL-28B rs8099917 genotypes among patients with acute hepatitis C was as follows: 37 (66.1%) were TT, 17 (30.4%) were GT and 2 (3.6%) were GG. Among chronic hepatitis C patients, 58(63.7%) were TT, 27 (29.7%) were GT and 6 (6.6%) were GG. We did not find any significant difference in the frequency of rs8099917 genotype based on liver disease status (P=0.7), but there was a significant difference in the frequency of rs12979860 genotype between acute and chronic HCV infected patients (P=0.001). 

## Discussion

Before initiation of antiviral therapy and in order to estimate the potential for successful treatment, the identification of parameters for the prediction of Sustained Virologic Response (SVR) in patients with chronic hepatitis C such as IL-28B SNPs as the strongest baseline predictor of SVRare important. They can help clinicians to decide whether or not to start antiviral therapy. They can also motivate patients who might have a high chance for virologic response. The different distributions of IL-28B genotypes in Asians, and Africans explain the different rates of SVR in these populations; therefore the determination of the IL-28B SNPs distribution is useful. In the present study, the most common IL-28B rs12979860 genotype in hepatitis C patients and healthy individuals was CT followed by CC and TT. Furthermore, the most prevalent rs8099917 genotype was TT, GT and GG respectively. The several studies in HCV infected patients in Europe, United State, Australia and Iran, as well as the recent study, showed that the most common rs12979860 genotype was CT, CC and TT consequently (20, 23, 24). The most frequency of rs8099917 genotype was TT followed by GT and GG, that similar to former studies (20, 25). Only few data are known about the frequency of IL-28B genotypes in the Middle East countries like Iran. Due to the low use of the IL-28B genotyping in the monitoring protocol of treatment, this report helps to better track the treatment in this region of Iran. 

Rs12979860 CC genotype is the favorable genotype for the strange prediction of response rate to PEG-IFN and RBV in patients who are infected with HCV genotype 1 or 4. In this study there was no difference in the frequency of rs12979860 and rs8099917 genotypes between total hepatitis C patients and the healthy individuals that is similar to other studies (20). However, a significant difference was seen if the healthy group was compared with genotype 3a infected patients, but the frequency of rs12979860 CC in patients infected with HCV genotypes 3a was lower than the healthy group. The frequency of rs12979860 CC in patients who were infected with HCV genotypes 3a was higher than those with HCV genotypes 1. This finding is in agreement with McCarthy et al. findings which showed rs12979860 genotype CC was significantly more common in patients with genotype 3a than in patients with HCV genotype 1 or 4 (26) that the underlying mechanism for this finding is not clear. Whether genotype CC leads the individual to be more prone to infection with HCV genotype 3a or whether infection with HCV genotype 3a becomes more common. But two recent studies have reported that the frequency of rs12979860 CC genotype in patients infected with HCV genotype 1, was lower than patients with HCV genotype 3a, and the healthy group (18, 19). Also, there was no difference in the frequency of IL-28B genotypes between patients with genotype 3a and healthy individuals that is parallel to our data. Several previous studies in HCV infected patients with genotype 1 and 4 reported that the spontaneous clearance of HCV was higher in hepatitis C patients with rs12979860 CC genotype than other rs12979860 genotypes (27-29). As a result, it seems that in the acute phase of HCV infection with viral genotypes 1 or 4, those who have IL-28B rs12979860 CC genotype clear the virus better than rs12979860 other genotypes. Therefore, in the chronic hepatitis C with HCV genotype 1, rs12979860 CC genotype is seen in lower frequency than the healthy group. However, in our study rs12979860 CC genotype frequency that related to HCV genotype 1 infected patient was similar to healthy individuals. Also, these studies showed rs12979860 CC genotype prevalence in acute hepatitis C patients was higher than chronic hepatitis C patients. Therefore, IL-28B polymorphisms probably have a significant role in the spontaneous clearance of HCV genotype 3 infected patients which results in the observation of the same distribution of IL-28B CC genotypes in the HCV genotype 1 infected patients and healthy individuals; of course the frequency of IL-28B genotypes in the HCV genotypes is suspicious in other studies. Also, HCV infected patients along with C alleles (CT, CC) may clear the virus better in acute HCV patients who are infected with genotype 3 than those with chronic hepatitis C. Similar results were seen in chronic hepatitis C patients who have been treated with a combination of Peg-IFN and RBV. It has been shown that among HCV infected patients with genotypes 1 or 4, those with rs12979860 CC genotype have a higher response rate to combination therapy than T allele carriers (30) while, another study showed a poor effect of IL-28B rs12979860 SNP on response rate to combination therapy in patients who were infected with genotype 2 and 3 (31). Our study showed IL-28B rs8099917 SNP, the frequency of rs8099917 TT genotype in patients infected with genotype 1 and 3a is higher than healthy individuals. Also, the frequency of rs8099917 GG genotype in patients infected with genotype 3a is higher than healthy individuals. In previous studies, the high frequency of the rs8099917 G allele in HCV genotype 1- or 4-infected patients suggest that the rs8099917 TT genotype may have a protective effect in terms of preventing the persistence of these two HCV genotypes (14). Therefore the different frequencies of the rs8099917 G allele in patients infected with different HCV genotypes may indicate that the innate immune system interacts differently with the different HCV genotypes. In our study rs8099917 GG genotype in patients infected with HCV genotype 3a may clear the virus better in patients with acute HCV than those with chronic hepatitis C and other genotypes.

The distribution of rs12979860 genotypes and alleles according to the HCV genotypes was notable. In conclusion, according to the proven role of the C allele in association with HCV treatment response and the spontaneous clearance of HCV, our data suggested that rs12979860 SNP may act differently in the clearance process of different HCV genotypes. Also the C allele can predict the spontaneous clearance rate of HCV in patients infected with different genotypes. Also, it seems in the acute phase of HCV infection with viral genotypes 3, those who have IL-28B rs12979860 CC genotype clear the virus better than rs12979860 T allele carriers. Therefore, rs12979860 CC genotype is seen in lower frequency in the chronic hepatitis C patients with HCV genotype 3, than healthy individuals. Finally, whether genotype CC leads the individual to be more prone to infection with HCV 3a genotype or opposite, therefore infection that caused by other genotypes in patients with rs12979860 CC are topics to be investigated. Therefore, further experimental investigations are needed involving the interaction between HCV genotype and IL-28B polymorphisms. 
